# Persisting Hypercalcemia and Hyperparathyroidism after Kidney Transplantation Have a Negative Impact on Graft and Patient Survival

**DOI:** 10.3390/diagnostics14131358

**Published:** 2024-06-26

**Authors:** Hannes Egli, Naomi Burla, Eva Breuer, Camilla Baron, Kerstin Hübel, Olivier de Rougemont, Harald Seeger, Diana Vetter

**Affiliations:** 1 Faculty of Medicine, University of Zurich, 8006 Zurich, Switzerland; hannes_egli@hotmail.com (H.E.); naomiburla@bluewin.ch (N.B.); 2 Department of Visceral and Transplant Surgery, University Hospital Zurich, 8032 Zurich, Switzerland; eva.breuer@usz.ch (E.B.); camilla.baron@usz.ch (C.B.); olivier.derougemont@hin.ch (O.d.R.); 3 Department of Nephrology, University Hospital Zurich, 8091 Zurich, Switzerland; kerstin.huebel@usz.ch (K.H.); harald.seeger@ksb.ch (H.S.)

**Keywords:** kidney transplantation, tertiary hyperparathyroidism, hyperparathyrodism, hypercalcemia

## Abstract

Hyperparathyroidism (HPT) with hypercalcemia, often deemed irreversible and detrimental to graft survival post-kidney transplantation (KT), prompts pre-transplant parathyroidectomy in hypercalcemic patients. In this retrospective analysis of 1212 kidney transplant recipients (KTRs) between 2006 and 2019, the incidence and effect of persistent HPT and hypercalcemia on graft and patient survival, and risk factors for persistence were analyzed until 60 months of follow up (FU). At KT, 5.7% (n = 69) had no HPT, 32.7% (n = 396) had HPT without hypercalcemia and 37.0% (n = 448) had HPT with hypercalcemia. At 2 years FU, 26.4% (n = 320) of patients had no HPT and 6% (n = 73) had HPT with hypercalcemia. Dialysis and dialysis duration were linked to HPT development, while dialysis, KT waiting time and donor type correlated with persisting hypercalcemia after KT. KTRs with normalized PTH and recovered hypercalcemia had improved death-censored graft survival (*p* < 0.001) and overall patient survival (*p* < 0.001). HPT with hypercalcemia is frequent at time of KT with normalization of PTH and calcium in a substantial proportion of patients after a KT. These findings question the routine pre-KT parathyroidectomy for suspected parathyroid autonomy. Persisting HPT, especially with hypercalcemia, adversely affects graft and patient survival, suggesting the need for more aggressive treatment of HPT, especially in cases of persisting hypercalcemia.

## 1. Introduction

Chronic kidney disease-mineral and bone disorder (CKD-MBD) is a complex syndrome in patients with chronic renal failure encompassing renal osteodystrophy, disturbances of mineral metabolism and cardiovascular disease [1]. Hyperparathyroidism (HPT) is a major feature of CKD-MBD. Depending on the CKD stage, the prevalence of HPT ranges from 20 to 80% [2,3,4]. HPT contributes to renal osteodystrophy, increases the risk for coronary artery disease [5,6] and is associated with high morbidity and mortality in hemodialysis patients [7,8,9,10].

In advanced chronic kidney disease, hyperphosphatemia, decreased extracellular calcium and decreased 1,25 (OH) Vit D3 lead to continuous stimulation of the parathyroid glands via direct and indirect mechanisms resulting in increased parathyroid hormone (PTH) [2] production and release [11].

In patients with improved renal function after kidney transplantation, abnormalities of calcium, phosphorus and vitamin D metabolism often normalize and PTH levels can gradually return to physiological levels. However, long-term stimulation of the parathyroid glands in CKD can lead to gland hyperplasia and with time, further polyclonal stimulation may result in nodular remodeling. From here, persistent activation can result in predominantly monoclonal proliferation with reduced sensitivity of the calcium-sensing and vitamin D receptors on the parathyroid cells. At this stage, parathyroid cells no longer underlie normal regulation and PTH rises further because calcium levels do not provide sufficient negative feedback. Consequently, serum calcium levels begin to rise indicating that parathyroid autonomy has developed. Currently, it is assumed that in these individuals, normalization of kidney function with renal transplantation does not lead to normalization of parathyroid function. HPT has been shown to decrease from 70% at one year to 43% at two years after KT [12]. Persistent HPT after KT has been found to have a negative impact on bone quality [13,14], graft survival [15] and increase mortality [15]. Parathyroidectomy (PTx) for HPT resulted in improved kidney graft function [16] if it was performed before or more than one year after kidney transplantation [16].

Only a few studies addressing the clinical impact of persistent HPT included calcemia in their analyses [14,15]. Perrin et al. found serum calcium levels to be significantly higher at the 12- and 60-month follow up in KTR that developed fractures [14]. Further, in a small series of patients with HPT and hypercalcemia, PTx was shown to improve bone density [17] and symptoms [18].

The goal of this study was to assess the course of HPT with and without hypercalcemia after KT and address its impact on graft and patient survival at a single tertiary center.

## 2. Materials and Methods

### 2.1. Data Set

Data from 1212 patients with kidney transplantation performed at the University Hospital of Zurich between January 2006 and September 2019 were analyzed.

### 2.2. Patient Characteristics

Sex, age at transplantation, dialysis status and dialysis vintage, as well as the time on the waiting list, type of KT (living donation or deceased donation as well as first- or re-transplantation), and information on cold and warm ischemia were assessed. Kidney function, presence of HPT and hypercalcemia were assessed by lab values (creatinine, eGFR using the CKD-EPI formula, PTH, calcium, phosphate, 25-OH Vitamine D3) at the time of the KT, and at different time points during the follow up (6, 12, 24, 48, and 60 months after KT, and at the last follow up) [9]. Clinical data such as graft and patient survival were collected.

If patients were treated by PTx, the information on the time point of PTx in relation to KT, as well as data on the specific surgical procedure and imaging were assessed.

### 2.3. Exclusion Criteria

The Swiss Ethics Committee on research involving human data approved the execution of this study (approval number: 2019-01848). Patients were excluded if no informed consent was available. Patient data was de-identified at time of retrieval from the electronic patient data management system.

### 2.4. Parathormone and Calcium Levels

Cutoff of elevated PTH was set at >65 ng/L and for albumin-corrected calcium >2.55 mmol/L in accordance with the clinic’s in-house laboratory protocols.

### 2.5. Kidney Function

eGFR was calculated using the Chronic Kidney Disease Epidemiological Collaboration (CKD-EPI) equation published in 2009 [19,20].

Primary non-function (PNF) was defined as graft loss within 3 months after KT [21].

### 2.6. Statistical Analysis

Baseline characteristics of numerical variables are reported as mean and standard deviation for variables with symmetric distribution and as a median and interquartile range for variables with asymmetric distribution. Comparison of numerical variables is carried out with the Student’s *t* test or Mann–Whitney test. Categorical variables are reported with counts and percentages and compared using the Fisher exact or the Pearson chi-squared test. Two-way ANOVA with multiple comparison was used to compare laboratory values over time. Where normality assumption is not met, the Kruskal–Wallis test is used. Time-to-event endpoints were visualized with Kaplan–Meier plots and Simon–Makuch survival plots with the status of HPT considered in a time-dependent way. Univariate and multivariable binary logistic regression analysis was used to assess for risk factors. All statistical tests are two-sided, and a *p* value < 0.05 is considered to be statistically significant. Statistical analyses and visualization were performed using R Statistical Software (Version 4.0.2; R Foundation for Statistical Computing, Vienna, Austria) and GraphPad Prism (version 8, San Diego, CA, USA).

## 3. Results

### 3.1. Patient Demographics

Of the 1212 transplantations, 364 (30%) were living-donor transplantations and 848 (70%) were deceased-donor transplantations (Table 1). The median age at transplantation was 52.1 years (IQR 39.6–60.7) and 62.3% of KTRs were men. Most KTRs had a first KT (n = 1038, 85.6%), whereas 12.5% (n = 152) had a re-transplantation and 10 patients (1.7%) had a re-re-transplantation. The median follow up was 62 months (IQR 28–105). KTRs with living donations were younger (*p* < 0.001), less often on dialysis at the timepoint of transplantation (*p* < 0.001) and if on dialysis, dialysis vintage was shorter than for deceased-donor KTRs (*p* < 0.001). The cold ischemia time was significantly shorter in living donor KTRs and the follow up was longer (*p* < 0.001) (Table 1).

### 3.2. Natural History of Hyperparathyroidism from Transplantation to Last FU

#### 3.2.1. High Rates of Secondary Hyperparathyroidism with and without Hypercalcemia at Timepoint of Transplantation

At transplantation, only 5.7% (n = 69) of patients had normal PTH values (Table 2), whereas 32.7% (n = 396) had secondary HPT without hypercalcemia and 37.0% (n = 448) had secondary HPT with hypercalcemia. At this timepoint, data was missing in 15.8% (n = 192) and incomplete in further 8.8% (n = 107) of patients (Table 2). Looking at a subset of 605 patients with complete information on calcium and PTH at the time of kidney transplantation and at the 2 year follow up where the rates were similar: no HPT was present at the time of transplantation in 7.1% (n = 43), secondary HPT in 39.3% (n = 238) and secondary HPT with hypercalcemia in 53.6% (n = 324) of patients before KT.

#### 3.2.2. A High Rate of Transplant Recipients with Hypercalcemia at Transplant Show Normalization of PTH and Calcium after Transplantation

Most patients without HPT at the time of transplantation kept a normal PTH after the transplantation. Normal PTH at KT as a result of prior PTx was seen in only 11 of 67 PTx patients.

**Figure 1:**
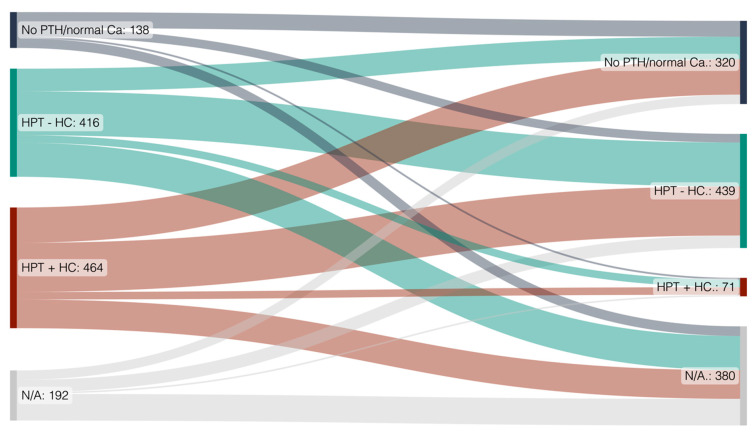
Sankey diagram of the whole KTR cohort and their calcium and PTH development over time. Left: at kidney transplantation, right: at 24-month follow up. Diagram created using SankeyMATIC.

In patients with secondary HPT and normal serum calcium at transplantation, PTH normalized in roughly one third of patients after transplantation, about 50% remained elevated but with normal serum calcium levels, and around 15% developed de novo hypercalcemia (Figure 1).

In a high rate of patients with secondary HPT and hypercalcemia at the time of transplantation, either both PTH and calcium normalized (no HPT), or calcemia alone normalized with persistently elevated PTH (Figure 1). Only a very small subset of patients remained hypercalcemic after transplantation. We observed similar findings in the subset of 605 patients with complete laboratory follow up at a timepoint of 24 months (Supplemental Figure S1).

Looking at the entire dataset, the rate of patients without HPT at the transplant increased from 5.7% (n = 69) at the time of KT to 21.5% (n = 261) at the 2-year follow up. The rate of patients with secondary HPT and no hypercalcemia increased from 32.7% (n = 396) to 36.1% (n = 437), and the percentage of patients with secondary HPT with hypercalcemia decreased from 37.0% (n = 448) to 4.5% (n = 55) after two years (Table 2).

### 3.3. Medication History with Potential Impact on Calcemia

#### 3.3.1. Vitamin D and Vitamin D Analogs

Vitamin D and analogs were prescribed very frequently (62.5–88.6%) in all patients at the KT, as well as at the 12- and 24-month follow up, independent of PTH-levels or the presence of hypercalcemia (Supplemental Figure S2). At the KT, only around one quarter of patients with hyperparathyreoidism had active Vitamin D (1,25(OH)_2_ vitamin D_3_) or 1,25(OH)_2_ vitamin D_3_ analogs in the form of calcitriol or paricalcitol, whereas 39.6% of HPT patients with hypercalcemia and 47.4% without hypercalcemia received inactive vitamin D (25(OH) vitamin D_3_, cholecalciferol). At the 12- and 24-month follow up, only a small percentage of patients had active vitamin D or analogs (1.9–4.1%). In patients with persisting HPT and hypercalcemia, 60.8% of patients had inactive vitamin D at 12 months and 59.7% at the 24-month follow up (Supplemental Figure S2).

#### 3.3.2. Calcimimetics

Calcimimetics were prescribed in 5.1–7.3% of patients at the time of transplantation. In patients with persisting hypercalcemia at the 12- and 24-month follow ups, calcimimetics were given in 14.4 and 15.3% of patients, respectively. In patients with persistent PTH-elevation but no hypercalcemia, calcimimetics were given in 4.9% at 12 months and 4.6% of patients at the 24-month follow up (Supplemental Figure S2).

### 3.4. Risk Factors for Hyperparathyroidis- and Hypercalcemia Development and Persistence after Kidney Transplantation

Risk factors for developing HPT before KT were dialysis and dialysis vintage. In addition, patients without a dialysis history developed less de novo HPT after the KT.

Regarding HPT with hypercalcemia, the incidence at KT was significantly higher in patients without a dialysis history. However, the rate of hypercalcemia normalization after KT was significantly higher in patients without previous dialysis, as well as in patients with shorter dialysis vintage, and shorter times on the KT waiting list (Supplemental Table S1). For example, patients with HPT and hypercalcemia at the time of the transplant that recovered from hypercalcemia after KT had a lower median time on dialysis (22 months, IQR 10–39) compared to those with persisting hypercalcemia at 24 months after the transplant (48.5 months, IQR 21.25–79.75). Similar findings were observed in the complete dataset.

### 3.5. Role of Parathyroidectomy in the Kidney Transplant Cohort

In 67 of the 1212 KTRs, HPT was treated surgically by PTx before KT. This subset of patients was on dialysis significantly longer and correspondingly had longer waiting times for the KT, as well as a higher rate of re-KTs (Supplemental Table S2). Their PTH values after transplantation were significantly lower than the rest of the cohort, but no differences in GFR were observed (Supplemental Table S2). There were no significant differences in graft and overall survival of the patients treated with PTx compared to the rest of the cohort (Supplemental Table S2).

### 3.6. Hyperparathyroidism in Living versus Deceased Kidney Donor Recipients and Its Effect on Patient and Graft Survival

#### 3.6.1. Parathyroid Hormone Is Lower in Patients after Living Compared to Deceased Kidney Donation despite Comparable Graft Function

**Figure 2:**
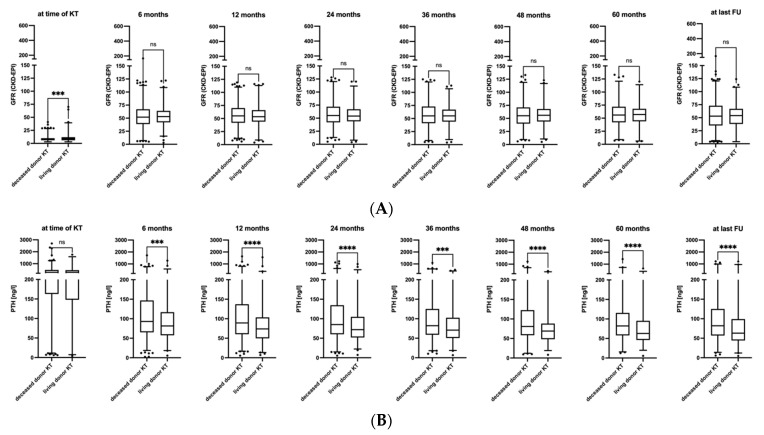
Comparison of GFR (**A**) and PTH (**B**) at kidney transplantation and all follow-up timepoints according to donor type. ***: *p* < 0.001, ****: *p* < 0.0001, ns: not significant. Black dots represent individual outlier cases outside the 95th percentile.

eGFR at transplantation was significantly lower in deceased donor KTRs than in living donor KTRs, but was no longer different between both groups after KT. The PTH levels on the other hand did not differ between both groups before transplantation, but—unlike equal post-transplantation kidney function—were significantly lower in living donation KTRs at all follow-up time points (Figure 2A,B).

#### 3.6.2. Living Donor KTRs Have a Higher Rate of PTH Normalization and an Overall Lower Rate of HPT with Hypercalcemia Than Deceased Donor KTRs (after KT)

At transplantation, living donor KTRs had a significantly higher rate of normal PTH and a higher rate of HPT with hypercalcemia than deceased donor KTRs (Supplemental Figure S3A,B). After transplantation, both groups showed an increase in patients with normal PTH and a decrease in patients with hypercalcemia. However, living donor KTRs showed a significantly higher rate of PTH normalization and an overall significantly lower percentage of patients with HPT and hypercalcemia at all follow-up time points (Supplemental Figure S3A,B). This association of donor type with development of HC on univariate analysis (*p* < 0.001) was, however, no longer significant in the multivariable binary logistic regression analysis when different possible hypercalcemia-inducing cofactors were included. Time on dialysis (*p* = 0.020) and waiting time for KT (*p* = 0.014) remained significant (Table 3). Similar results were found for the complete dataset where living donor KTRs had a higher rate of PTH normalization at the 2-year follow up (58.4% vs. 24.1%). Time on dialysis was shorter for patients with PTH normalization (36 mo vs. 40 mo, ns), albeit not significant, and the time on the waiting list was significantly shorter (19 mo vs. 29 mo, *p* < 0.001) for patients with PTH normalization.

#### 3.6.3. Living Donor KTRs Have the Same Death-Censored Graft Loss, but Overall Better Survival than Deceased Donor KTRs

Living donor KTRs had a significantly longer overall patient survival (HR: 1.93 (95% CI 1.35–2.77), *p* < 0.001), (Supplemental Figure S4A). Although the risk for graft loss in living donor KTRs was significantly lower (HR: 1.53 (95% CI 1.14–2.05), *p* = 0.004) (Supplemental Figure S4B), death-censored graft loss, showed no significant difference between the groups (HR: 1.18 (95% CI 0.76–1.85), *p* = 0.461), (Supplemental Figure S4C).

Both, dialysis and waiting time for transplants were negative predictive factors for patient survival and graft loss, but not for death-censored graft loss in the Cox regression analysis.

### 3.7. PTH Normalization after Kidney Transplantation Has a Positive Association with Graft and Overall Survival

**Figure 3:**
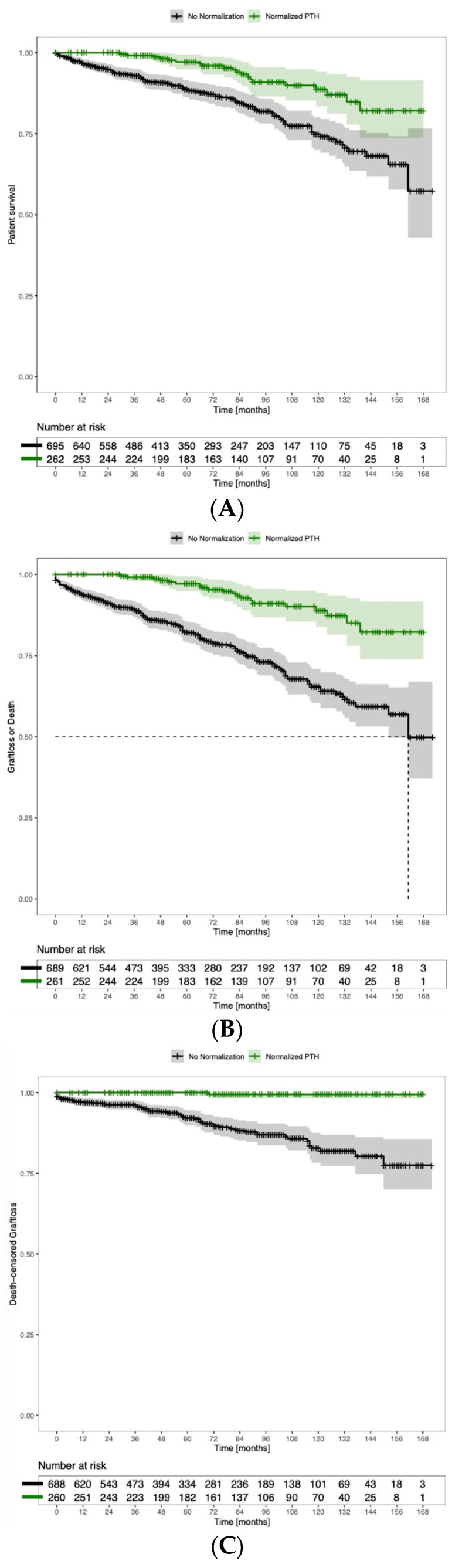
(**A**) Patient survival according to normalization of PTH. Logrank (Mantel–Cox): HR 2.61, 95% CI 1.62–4.31, *p* < 0.001. (**B**) Graft survival according to normalization of PTH. Logrank (Mantel–Cox): HR 3.88, 95% CI 2.43–6.19, *p* < 0.001. (**C**) Death-censored graft survival according to normalization of PTH. Logrank (Mantel–Cox): HR 32.67, 95% CI 4.53–235.51, *p* < 0.001.

Patients with normalized PTH after KT showed a significantly better kidney function at all follow-up timepoints (Table 4). Overall patient survival was significantly improved in the KTRs with normalized PTH after transplantation (HR 2.61, 95% CI 1.62–4.31, *p* < 0.001), (Figure 3A).

Further, both the overall graft survival (HR 3.88, 95% CI 2.43–6.19, *p* ≤ 0.001), and the death-censored graft survival (HR 32.67, 95% CI 4.52–235.51, *p* < 0.001) were significantly higher in the subset of patients with PTH normalization (Figure 3B,C).

Similar results were found in the complete dataset: median GFR at the 24-month follow up was significantly higher in the PTH-normalized patients (60 mL/min vs. 53 mL/min, *p* < 0.001), and patients with normalized PTH had significantly better overall survival (*p* = 0.031), less graft loss (*p* = 0.002) and less death-censored graft loss (*p* = 0.002).

### 3.8. Persisting Hypercalcemia Is Associated with Negative Graft and Overall Survival

**Figure 4:**
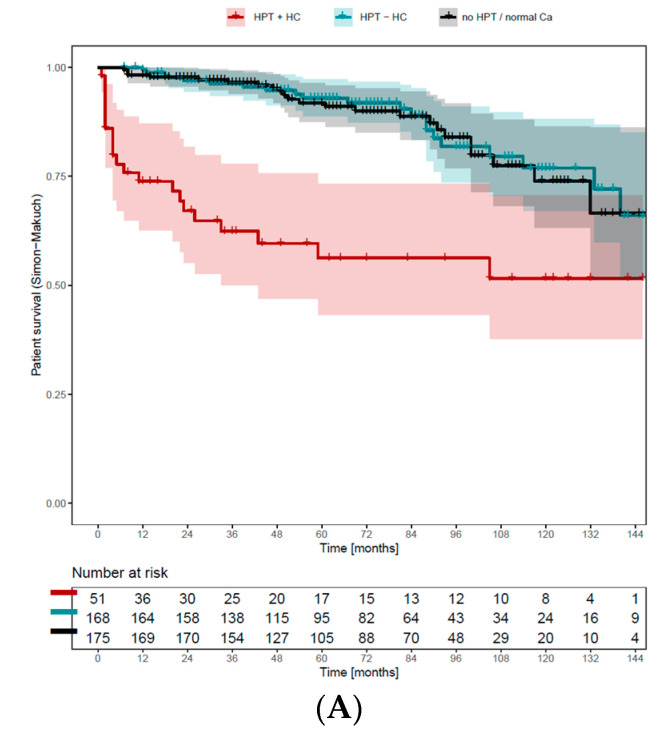

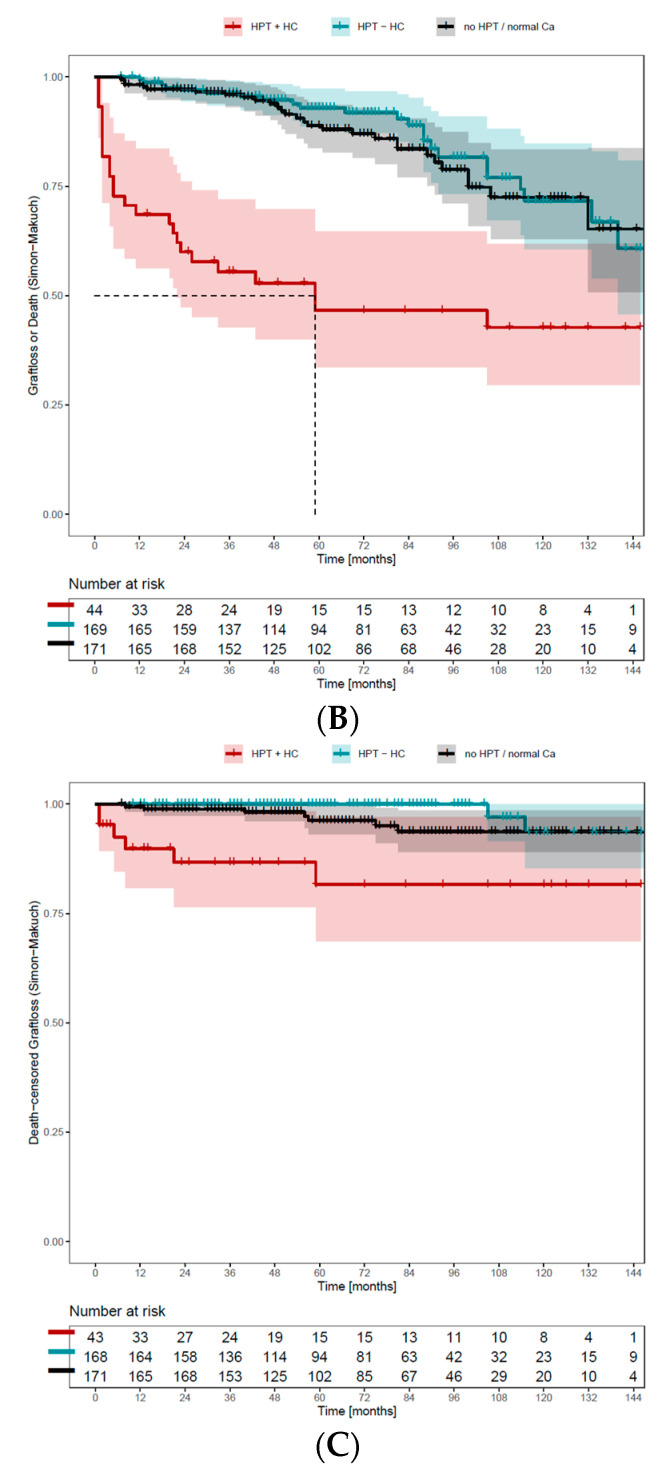
Simon–Makuch plot for overall survival (**A**), graft loss (**B**), and death-censored graft loss (**C**) with status of hyperparathyroidism as a time-dependent covariate. At time 0 (kidney transplantation), all patients are in the group “HPT + autonomy” (n = 466). Upon a change in the status, each individual patient is removed from the risk set in the group “HPT + autonomy” and added to the risk set of either “HPT–autonomy” or “no HPT”, in which he or she remains until death, graft loss or death-censored graft loss. Here, the patients that recovered display better overall survival (HR 2.08, 95% CI 1.05–4.17 *p* = 0.036), less graft loss (HR 2.22, 95% CI 1.19–4.17, *p* = 0.013) and less death-censored graft loss (HR 3.23, 95% CI 0.88–12.5, *p* = 0.078) compared to those with persisting hypercalcemia after kidney transplantation.

When considering only the patients with HPT and hypercalcemia at the timepoint of KT (n = 466), those with persisting hypercalcemia after KT displayed significantly worse patient and graft survival compared to those without hypercalcemia or normalized HPT (Figure 4A–C). Survival data was analyzed including the change of HPT status after KT in a time-dependent way using Simon–Makuch plots. Patients with persisting hypercalcemia during the follow up showed a significantly worse graft (HR 2.22, 95% CI 1.19–4.17, *p* = 0.013) and overall survival (HR 2.08, 95% CI 1.05–4.17, *p* = 0.036), as well as worse death-censored graft loss (HR 3.23; 95% CI 0.88–12.5; *p* = 0.078), although this was only borderline significant.

Factors associated with persisting hypercalcemia were dialysis status, dialysis type, time on waiting list, as well as donor type (Supplemental Table S3).

## 4. Discussion

This study on a large cohort of KTRs confirms the high rate of HPT at transplantation and underlines the importance of PTH normalization on kidney graft and overall patient survival. Our study provides two new important insights. First, the observation of a high hypercalcemia recovery rate from 37% at transplant to 4.5% at the 2-year follow up that can be interpreted as autonomy reversal. The second finding is the significant negative association with graft and overall survival if hypercalcemia persists long term after KT.

The low rate of patients without HPT at the time of transplantation of around 6% is similar to other studies where 90% of patients with kidney failure were found to have HPT at KT [3]. However, the HPT normalization rate observed in the current work is lower than in previous studies [12,22]. For example, two years after KT, only 21.5% of patients had a normalized PTH, compared to 56.9% in another series [12]. This may be due to a higher rate of living donor transplantations (42.5% [12], or 43% [22] versus 30%) and shorter dialysis vintage in the previous studies [12].

In previous studies, hypercalcemia incidence after KT has been reported with high variability from <5% up to >50% [23,24,25,26,27,28,29,30]. Whereas most studies describe a low rate of HC at KT that decreases within the first months and remains around 15%, findings in our study are similar to the observations in Reinhardt et al. [23], with high hypercalcemia rates at the time of KT (37 versus 52% [23]), and a significant drop (4.5% vs. 10% [23]) at the 2-year follow up. The major risk factor for developing hypercalcemia after KT is thought to be a severe form of HPT due to long-lasting CKD. Consequently, the reason for the substantial variability of HC incidence after KT may lie in different listing policies with consecutive differences in waiting times until kidney transplantation.

To date, the presence of parathyroid gland autonomy in patients with renal HPT was thought to be irreversible. Thus, if present in renal transplant candidates, PTx before transplantation was considered in order to protect the kidney graft from hypercalcemia [16]. The advantage of PTx prior to transplantation in comparison to after transplantation on graft function was demonstrated by Littbarski et al. [16].

The finding that up to 37% of KTRs have HPT with HC at the time of transplantation and that this decreases to below 10% at the two-year follow up, suggests reversibility of parathyroid autonomy, when the causal renal insufficiency is treated by transplantation. This finding could have been impacted by an intake of vitamin D in patients with CKD, as this may cause hypercalcemia. As 64.6% of KTRs with HPT and hypercalcemia took vitamin D analogs at the time of transplant, this may have led to a falsely high rate of hypercalcemia at kidney transplantation. However, only 25.1% of patients with HPT and hypercalcemia at the time of transplant took active vitamin D. The remaining 39.6% took inactive vitamin D, which is less likely to lead to hypercalcemia. On the other hand, intake of calcimimetics may have caused lowering of calcemia, thus masking patients with “true” hypercalcemia. Merely 5.5% and 6.8% of KTRs with non-elevated calcium levels were on calcimimetics at the 12- or 24-month follow up, so the intake of calcimimetics at the follow up should not relevantly mask the presence of autonomous parathyroid glands. Thus, the low rate of hypercalcemia at the follow up seems to be real.

If the high hypercalcemia rate at transplantation is due to the development of autonomy, the observed decrease in hypercalcemia at the follow up after transplantation would reflect autonomy reversibility. This may question the approach of routine early PTx prior to transplantation in KT candidates with HPT and suspected autonomy.

On the other hand, persisting hypercalcemia at the long-term follow up (24 months) had a significant negative impact on graft and overall survival of KTRs. Thus, detection and treatment of patients with persisting hypercalcemia seems to be important. As dialysis status, dialysis type, time on KT waiting list, and donor type were associated with persisting hypercalcemia at the long-term follow up, early PTx before KT may be advantageous for patients with hypercalcemia and these risk factors.

Risk factors for developing HPT, for PTH normalization and for recovery from parathyroid autonomy in the current study were deceased donation KT, being on dialysis before transplantation, dialysis vintage and time on the transplant waiting list. Dialysis vintage, and time on waiting list were also found to be factors associated with PTH normalization in previous studies [31,32]. The association of deceased-donation KT and the presence of parathyroid autonomy was no longer present in the multivariable logistic regression, indicating that the negative effect is indirect and possibly due to the higher percentage of patients on dialysis in this group, as well as dialysis vintage and longer times on the waiting list.

The small subset of patients in the cohort treated by PTx showed a significantly lower PTH at all follow-up timepoints than the rest of the cohort; however, this was without having a positive impact on graft and overall survival. This may be due to the small number of patients, and the higher morbidity in this subset of patients with significantly longer times on dialysis and on the KT waiting list, and a higher rate of re-transplantations than seen in the rest of the cohort. Thus, the higher cardiovascular morbidity may mask any positive effect that PTx may have on graft function.

Looking at the differences between living- and deceased-donation KT, PTH values were significantly lower at all follow-up time points in the living donor cohort. This is despite having comparable PTH levels before transplantation and having similar graft function. The fact that deceased and living donor recipients have comparable graft function is already known [33]. It is also known that mineral metabolism normalizes faster in living donor KTRs than in deceased donor KTRs [34]. Living donation KTRs also show an increased rate of patients with normal PTH to begin with, as well as a higher rate of normalization of HPT, in parts because of a significantly higher probability to lose parathyroid autonomy. This may again be due to the lower rate of patients on dialysis, shorter duration of dialysis or time on waiting list, which are all risk factors for developing de novo or maintaining persistent parathyroid autonomy.

To our knowledge, this is the first study to establish an improved HPT prognosis for living donations. In contrast to the current study, donor type was not significantly associated with HPT normalization in the multivariate analysis in the study from Lou et al. [12], possibly because waiting time on transplant list as well as time on dialysis were overall longer in the current study.

As the graft survival, but not the death-censored graft survival, was better in living donor KTRs, this indicates that it is not the better graft survival causing the improved survival, but that the living donor KTRs might be healthier than deceased donor KTRs to begin with. This again is well explained by the favorable setting at transplantation, overall younger age, and higher rates of patients with normal PTH.

The finding that PTH normalization is significantly associated with better graft survival, death-censored graft survival and overall survival, is in line with other studies [12,15,35,36]. Pathogenesis hereof is not clear, but vasoconstriction and tubulointerstitial calcification in patients with persistent HPT after KT are thought to be causes [36,37]. Further, HPT is a known cardiovascular risk factor.

To our knowledge, the finding that persistent hypercalcemia at long-term follow up is associated with worse kidney graft and overall survival is novel. The survival curves of patients not recovering from hypercalcemia are clearly different from those either fully recovering from HPT (=no HPT), or those with persisting HPT but without hypercalcemia, which have a similar course. This implies that the recovery from hypercalcemia could be an important factor positively affecting survival in these patients.

A limitation of the study is the incomplete dataset. Due to the retrospective nature of the study and the fact that after KT, the referring nephrologists outside of the University Hospital of Zurich continued treatment, data was not readily available for all KTRs in the University Hospital electronic data system.

## 5. Conclusions

Hyperparathyroidism with suspicion of autonomy is frequent at the time of KT. However, a substantial number of patients can fully normalize PTH and plasma calcium after KT, strongly questioning routine pre-KT parathyroidectomy for suspected parathyroid autonomy. Hyperparathyroidism, particularly with persisting hypercalcemia, is associated with worse graft and patient survival. Whether more aggressive treatment of HPT with persisting hypercalcemia is warranted needs to be addressed in future prospective trials.

## Figures and Tables

**Table 1:** Patient characteristics of the 1212 kidney transplant recipients.

	Total Cohort (n = 1212)	Deceased (n = 848)	Living Donors (n = 364)	*p*	Missing
Basic patient data					
Age at KT, median (IQR)	52.1 (39.6–60.7)	53.4 (41.9–62)	46.9 (34.5–57.7)	<0.001	-
Male gender, n (%)	755 (62.3)	527 (62.1)	228 (62.6)	0.872	-
FU-time (months), median (IQR)	62 (28–105)	57 (26–97)	79 (37–121)	<0.001	4.0%
Calcium at last FU, median (IQR)	2.32 (2.24–2.41)	2.32 (2.24–2.41)	2.31 (2.24–2.39)	0.447	47.9%
Creatinine at last FU, median (IQR)	121 (93–169.5)	121 (93–169.5)	124.5 (102–164.8)	0.153	33.9%
Phosphate at last FU, median (IQR)	0.93 (0.80–1.11)	0.93 (0.80–1.11)	0.95 (0.79–1.11)	0.972	41.7%
1,25-dihydroxyvitamin D, median (IQR)	32 (24.2–38.9)	32 (24.2–38.9)	32.8 (25.9–40.2)	0.255	69.5%
Transplantation related					
Time on waitlist (months), median (IQR) Dialysis before transplantation, n (%) No dialysis HD CAPD CCPD Other or combination Duration of dialysis (months), median (IQR) Number of KT, n (%)	22 (9–42) 231 (19.1) 810 (66.8) 162 (13.4) 2 (0.2) 4 (0.3) 33 (17–55)	28 (14–48) 94 (11.1) 629 (74.3) 120 (14.2) 2 (0.2) 2 (0.2) 39 (23–60)	8 (4–15) 137 (37.8) 181 (50.0) 42 (11.6) 2 (0.6) 0 (0.0) 12 (5–27)	<0.001 <0.001 <0.001 0.429	8.5% 0.2% 0.3% -
1	1038 (85.6)	718 (84.7)	320 (87.9)		
2	152 (12.5)	114 (13.4)	38 (10.4)		
3	20 (1.7)	15 (1.8)	5 (1.4)		
4	2 (0.2)	1 (0.1)	1 (0.3)		
Living-Donor KT Warm ischemia (min), median (IQR) Cold ischemia (h), median (IQR)	364 (30.0) 30 (20–36) 9.5 (7.1–12.3)	- 27 (17–37) 9.9 (7.4–12.5)	364 (100) 30 (30–35) 2.1 (2.1–2.3)	<0.001 0.038 <0.001	- 88.8% 27.1%
Rejection before organ failure, n (%)	91 (7.9)	63 (7.7)	28 (8.4)	0.728	5.3%
Time from TPL to rejection (months), median (IQR)	39 (4.3–69.9)	35.6 (1.7–66.5)	52.3 (15.9–77.7)	0.082	-
Primary non-function, n (%)	22 (1.8)	18 (2.1)	4 (1.1)	0.221	-
Patient and graft survival					
Patient survival, %					
1-year	96.6	95.6	98.8	<0.001	0.7%
2-year	95.1	94.0	97.9		
5-year	89.8	87.5	94.8		
10-year	77.2	74.2	83.5		
Graft survival, %					
1-year	94.2	92.9	97.6	0.004	2.3%
2-year	92.2	90.9	95.7		
5-year	84.7	82.5	90.0		
10-year	70.1	67.8	75.1		
Death-censored graft survival, %					
1-year	97.1	96.6	98.1	0.461	5.4%
2-year	96.5	96.2	97.2		
5-year	93.4	93.1	94.0		
10-year	87.2	86.6	88.3		

**Table 2:** Number and percentages of KTRs without hyperparathyroidism, with hyperparathyroidism and normo- or hypocalcemia, as well as KTRs with hyperparathyroidism and hypercalcemia at KT and various timepoints thereafter. Number of missing or incomplete data are also listed as N/A.

	No 2HPT	PTH not Available Normal Calcium	PTH not Available Hypocalcemia	2HPT Normo- or Hypocalcemia	PTH Elevated Calcium not Available	PTH not Available Hypercalcemia	PTH Elevated Hypercalcemia	N/A (missing data)
Tx	69 (5.7)	69 (5.7)	20 (1.7)	396 (32.7)	8 (0.7)	10 (0.8)	448 (37.0)	192 (15.8)
6 months	262 (21.6)	61 (5.0)	1 (0.1)	603 (49.8)	21 (1.7)	13 (1.1)	101 (8.3)	150 (12.4)
12 months	316 (26.1)	46 (3.8)	3 (0.2)	536 (44.2)	20 (1.7)	5 (0.4)	74 (6.1)	212 (17.5)
24 months	261 (21.5)	59 (4.9)	2 (0.2)	437 (36.1)	15 (1.2)	3 (0.2)	55 (4.5)	380 (31.4)
5 years	196 (16.2)	57 (4.7)	5 (0.4)	255 (21.0)	5 (0.4)	3 (0.2)	21 (1.7)	670 (55.3)
last FU	146 (12.0)	190 (15.7)	34 (2.8)	224 (18.5)	23 (1.9)	34 (2.8)	9 (0.7)	552 (45.5)

**Table 3:** Univariate and multivariable binary regression analysis of risk factors for developing hyperparathyroidism and hypercalcemia after kidney transplantation. Univariate and multivariable analysis of risk factors for developing 2HPT + HC.

	Univariate			Multivariable		
	*p*	HR	95% CI	*p*	HR	95% CI
Female sex	0.148	0.76	0.53–1.10	0.171	0.75	0.50–1.13
Donor age	0.798	1.00	0.99–1.01	0.988	1.00	0.99–1.01
Time on waiting list (months)	<0.001	1.02	1.02–1.03	0.014	1.01	1.00–1.02
Dialysis time (months)	<0.001	1.01	1.01–1.02	0.020	1.01	1.00–1.02
LDKT	<0.001	0.43	0.27–0.70	0.342	0.684	0.31–1.50

**Table 4:** The effect of PTH normalization on graft function at all follow-up timepoints after kidney transplantation. Association of PTH normalization with graft function.

	PTH not Normalized n = 696	PTH Normalized n = 263	*p*	Missing
GFR (mL/min), median (IQR)				
KT	8 (6–10)	8 (6–11)	0.076	6.2%
6 months	51 (37–64)	55 (44–68)	<0.001	4.2%
12 months	53 (40–67)	58 (48–73)	<0.001	8.7%
24 months	53 (39–67)	61 (48–79)	<0.001	21.8%
36 months	52 (39–66)	60 (47–76)	<0.001	33.1%
48 months	52 (38–66)	59 (49–76)	<0.001	42.6%
5 years	53 (38–68)	59 (50–75)	<0.001	51.0%

## Data Availability

The data presented in this study are available on request from the corresponding author.

## Supplementary Materials

The following supporting information can be downloaded at: https://www.mdpi.com/article/10.3390/diagnostics14131358/s1, Supplemental Table S1: Effect of pre-transplantation dialysis status on incidence and evolution of hyperparathyroidism and hypercalcemia in KTRs; Supplemental Table S2: Patient characteristics, and graft- and overall survival for patients with parathyreoidectomy before kidney transplantation compared to the rest of the transplant cohort; Supplemental Table S3: Patient characteristics and outcomes of KTRs without hypercalcemia-compared to KTRs with persisting hypercalcemia at 24 month FU or at death (if patients died before). Patient and graft survival data are derived from the Simon-Makuch survival analyses; Supplemental Figure S1: Sankey diagram of a subset of patients (n = 605) with complete data on serum calcium and serum PTH at the two observed timepoints. Left: at kidney transplantation, right: at 24 month follow-up. Diagram created using SankeyMATIC; Supplemental Figure S2: Overview of medical treatment, including Vitamin D, Vitamin D analogs and calcimimetics, at time of transplantation, 12 and 24 months after kidney transplantation; Supplemental Figure S3: 3A: Distribution of no HPT, HPT without hypercalcemia and HPT with hypercalcemia between living donor- and deceased donor KTRs at transplantation and all follow-up timepoints (from left to right: time of transplantation, 6 months, 12 months, 24 months and 60 months after transplantation); 3B: Distribution of no HPT, HPT without hypercalcemia and HPT with hypercalcemia between living donor- and deceased donor KTRs at transplantation and all follow-up timepoints in a subset of patients with complete dataset (from left to right: time of transplantation, 6 months, 12 months, 24 months and 60 months after transplantation); 3C: Incidence of hyperparathyroidism and hypercalcemia over all timepoints between living donor- and deceased donor KTRs; Supplemental Figure S4: 4A: Overall survival of patients according to donor type. HR:1.93 (95% CI 1.35–2.77), *p* < 0.001; 4B: Graft survival of patients according to donor type. HR:1.53 (95% CI 1.14–2.05), *p* = 0.004; 4C: Death-censored graft survival of patients according to donor type. HR:1.18 (95% CI 0.76–1.85), *p* = 0.461.
