# Supplementary material for: Persisting Hypercalcemia and Hyperparathyroidism after Kidney Transplantation Have a Negative Impact on Graft and Patient Survival
Source: Diagnostics (Basel). 2024 Jun 26;14(13):1358. doi: 10.3390/diagnostics14131358 (PMC11240723; doi:10.3390/diagnostics14131358)
Supplement: Supplementary file 1 [file diagnostics-14-01358-s001.zip › diagnostics-3011052-supplementary.pdf]

Supplemental Tables and Figures

Supplemental Table S1

Effect of pre-KT dialysis on the incidence and evolution of HPT and HC, n=1209 (missing dialysis status of n=3 patients)				
**At timepoint of Tx**	**Total cohort** (n=1212)	**No dialysis** (n=231)	**Dialysis** (n=978)	**p**
No PTH / normal Ca	137 (11.3) (13.5)	30 (13.0) (17.8)	107 (10.9) (12.6)	<0.001
2 HPT - hypercalcemia	416 (34.4) (40.9)	38 (16.5) (22.5)	378 (38.7) (44.5)	
2 HPT + hypercalcemia	465 (38.5) (45.7)	101 (43.7) (59.8)	364 (37.2) (42.9)	
N/A	191 (15.8) .	62 (26.9) .	129 (13.2) .	
**At 6 months**				
No PTH / normal Ca	322 (26.6) (30.4)	81 (35.1) (44.0)	241 (24.6) (27.5)	<0.001
2 HPT - hypercalcemia	602 (49.8) (56.8)	92 (39.8) (50.0)	510 (52.1) (58.3)	
2 HPT + hypercalcemia	135 (11.2) (12.7)	11 (4.8) (6.0)	124 (12.7) (14.2)	
N/A	150 (12.4) .	40 (20.3) .	103 (10.5) .	
**At 12 months**				
No PTH / normal Ca	361 (29.6) (36.2)	90 (39.0) (51.7)	271 (27.7) (32.9)	<0.001
2 HPT - hypercalcemia	537 (44.4) (53.9)	77 (33.3) (44.3)	460 (47.0) (55.9)	
2 HPT + hypercalcemia	99 (8.2) (9.9)	7 (3.0) (4.0)	92 (9.4) (11.2)	
N/A	212 (17.5) .	57 (24.7) .	155 (15.8) .	
**At 24 months**				
No PTH / normal Ca	319 (26.4) (38.4)	76 (32.9) (51.4)	243 (24.8) (35.6)	<0.001
2 HPT - hypercalcemia	438 (36.2) (52.8)	68 (29.4) (45.9)	370 (37.8) (54.3)	
2 HPT + hypercalcemia	73 (6.0) (8.8)	4 (1.7) (2.7)	69 (7.1) (10.1)	
N/A	379 (31.3) .	83 (35.9) .	296 (30.3) .	
**At 5 years**				
No PTH / normal Ca	252 (20.8) (46.6)	63 (27.3) (58.3)	189 (19.3) (43.6)	0.021
2 HPT - hypercalcemia	260 (21.5) (48.1)	43 (18.6) (39.8)	217 (22.2) (50.1)	
2 HPT + hypercalcemia	29 (2.4) (5.4)	2 (0.9) (1.9)	27 (2.8) (6.2)	
N/A	668 (55.3) .	123 (53.2) .	545 (55.7) .	

Supplemental Table S2

Patient Characteristics for patients with PTx before kidney transplantation (KT), n=67

	**Complete cohort without PTx** (n=1075)	**PTx before KT** (n=67)	**p**
**Basic patient data**			
Age at KT, median (IQR)	52.1 (41 – 61.3)	49.7 (40.8 – 59.5)	0.378
Male gender, n (%)	680 (63.3)	38 (56.7)	0.282
FU-time (months), median (IQR)	62 (30 – 108)	70 (37 – 103)	0.524
**Transplantation-related**			
Time on waitlist (months), median (IQR)	20.5 (8 – 40)	41 (11.5 – 69.5)	<0.001
Dialysis before transplantation, n (%)			0.638
No dialysis	198 (18.5)	8 (11.9)	
HD	734 (68.5)	47 (70.1)	
CAPD	135 (12.7)	12 (17.9)	
CCPD	1 (0.1)	0 (0.0)	
Other or combination	4 (0.4)	0 (0.0)	
Duration of dialysis (months), median (IQR)	32 (17 – 52)	59 (21 – 97)	<0.001
Number of Tx, n (%)			<0.001
1	931 (86.6)	41 (61.2)	
2	128 (11.9)	20 (29.9)	
3	14 (1.3)	6 (9.0)	
4	2 (0.2)	0 (0.0)	
Living-Donor Kidney Tx	323 (30.0)	22 (32.8)	0.630
Warm ischemia (min), median (IQR)	30 (19 – 36)	22 (20 – 30.5)	0.332
Cold ischemia (h), median (IQR)	9.6 (7.1 – 12.3)	9.4 (7.3 – 11.75)	0.946
Rejection before organ failure, n (%)	82 (7.7)	6 (9.1)	0.691
Time from TPL to rejection (months), median (IQR)	40.9 (5.3 – 71.8)	12.1 (1.7 – 77.7)	0.518
Primary non-function, n (%)	18 (1.7)	2 (3.0)	0.427
**PTx**			
Indication for PTx			-
3 HPT	-	0 (0.0)	
2 HPT + suspicion of autonomy	-	38 (56.7)	
2 HPT – suspicion of autonomy	-	26 (38.8)	
unknown	-	3 (4.5)	
**Patient and graft survival**			
Patient survival, %			0.733
1-year	96.6	96.9	
2-year	95.0	96.9	
5-year	89.5	93.1	
10-year	77.2	69.6	
Graft survival, %			0.684
1-year	94.6	92.3	
2-year	92.5	90.6	
5-year	84.6	86.9	
10-year	70.5	56.2	
Death-censored graft survival, %			0.666
1-year	97.4	95.4	
2-year	96.9	93.6	
5-year	93.5	93.6	
10-year	87.4	82.5	
**Lab values**			
GFR (ml/min), median (IQR)			
Tx	8 (6 – 11)	7 (5 – 10)	0.163
6 months	52 (40 – 66)	50 (37 – 60)	0.295
12 months	55 (42 – 69)	56 (43 – 68)	0.356
24 months	55 (42 – 70)	54 (42 – 69)	0.571
36 months	55 (42 – 70)	58 (38 – 68)	0.675
48 months	55 (42 – 70)	51 (33 – 72)	0.323
5 years	56 (44 – 71)	54 (35 – 74)	0.328
PTH (ng/l), median (IQR)			
Tx	285.9 (168.7 – 481.6)	77.7 (21.3 – 283.2)	<0.001

6 months	90 (64.2 – 134.1)	80.6 (34.9 – 161.7)	0.083
12 months	84.5 (58.7 – 123)	61.6 (29.5 – 143.1)	0.012
24 months	81.7 (58 – 122.9)	58 (25.6 – 135.1)	0.019
36 months	77.7 (57 – 115.9)	63.9 (31.9 – 135.4)	0.064
48 months	78.2 (57.1 – 110.4)	45.7 (23.3 – 79)	<0.001
5 years	75.8 (54.8 – 110.1)	44.6 (24.8 – 75.1)	<0.001

Supplemental Table S3

Patient Characteristics, n=466; *censoring when before				
**Patient and graft survival**	**no HC at 24 months or at timepoint of death*** (n=395)	**2HPT+ HC at 24 months or at timepoint of death*** (n=71)	p	missing
Patient survival, %				
1-year	99.0	77.1	<0.001	0.6%
2-year	97.6	71.9		
5-year	92.6	63.9		
10-year	75.7	54.8		
Graft survival, %				
1-year	97.9	68.2	<0.001	1.1%
2-year	96.3	61.8		
5-year	89.6	54.7		
10-year	70.7	46.8		
Death-censored graft survival, %				
1-year	99.0	85.3	<0.001	2.1%
2-year	98.7	83.1		
5-year	96.9	83.1		
10-year	92.1	83.1		
**Transplantation-related**				
Time on waitlist (months), median (IQR)	19 (7 – 38)	25 (8.3 – 53)	0.036	4.1%
Type of donation			0.034	0.0%
Living donors	140 (35.4)	16 (22.5)		
Deceased donors	255 (64.6)	55 (77.5)		
Dialysis before transplantation, n (%)	300 (75.9)	64 (90.1)	0.008	0.2%
Type of dialysis, n (%)			0.039	0.0%
No dialysis	94 (23.8)	7 (9.9)		
HD	246 (62.3)	56 (78.9)		
CAPD	54 (13.7)	8 (11.3)		
CCPD	0 (0.0)	0 (0.0)		
Other or combination	0 (0.0)	0 (0.0)		
Duration of dialysis (months), median (IQR)	32 (16 – 53)	39 (14.8 – 67)	0.166	22.1%
**Lab values**				
GFR (ml/min), median (IQR)				
KT	8 (6 – 11)	8 (6 – 10)	0.240	0.6%
6 months	52 (41 – 64)	51 (35 – 67)	0.999	4.1%
12 months	54 (42 – 66)	56 (43 – 75)	0.419	8.2%
24 months	55 (42 – 70)	58 (42 – 69)	0.831	20.6%
36 months	55 (42 – 69)	58 (46 – 73)	0.468	33.7%
48 months	55 (41 – 68)	59 (47 – 76)	0.294	45.3%
5 years	56 (42 – 70)	60 (53 – 75)	0.363	53.6%
Last FU	54 (39 – 70)	53 (9 – 72)	0.176	2.4%
PTH (ng/l), median (IQR)				
KT	288.8 (176 – 474.9)	285.8 (208.4 – 538.3)	0.427	4.1%
6 months	91.4 (66.7 – 134.2)	169.9 (106.8 – 254.7)	<0.001	12.7%

12 months	83 (58 – 121.5)	156.2 (102.9 – 216.1)	<0.001	15.7%
24 months	75.3 (56.7 – 120.5)	136.7 (103.7 – 179.1)	<0.001	29.0%
36 months	69.8 (53.7 – 115.6)	134.9 (101.4 – 179.6)	<0.001	42.5%
48 months	72.8 (52.9 – 105.1)	110.8 (62.7 – 158.2)	0.014	52.8%
5 years	72.3 (50.3 – 109.4)	91.4 (60 – 139.3)	0.223	59.9%
Last FU	71.3 (51.4 – 109.8)	142.5 (60 – 273.2)	0.002	46.4%

Supplemental Figure S1

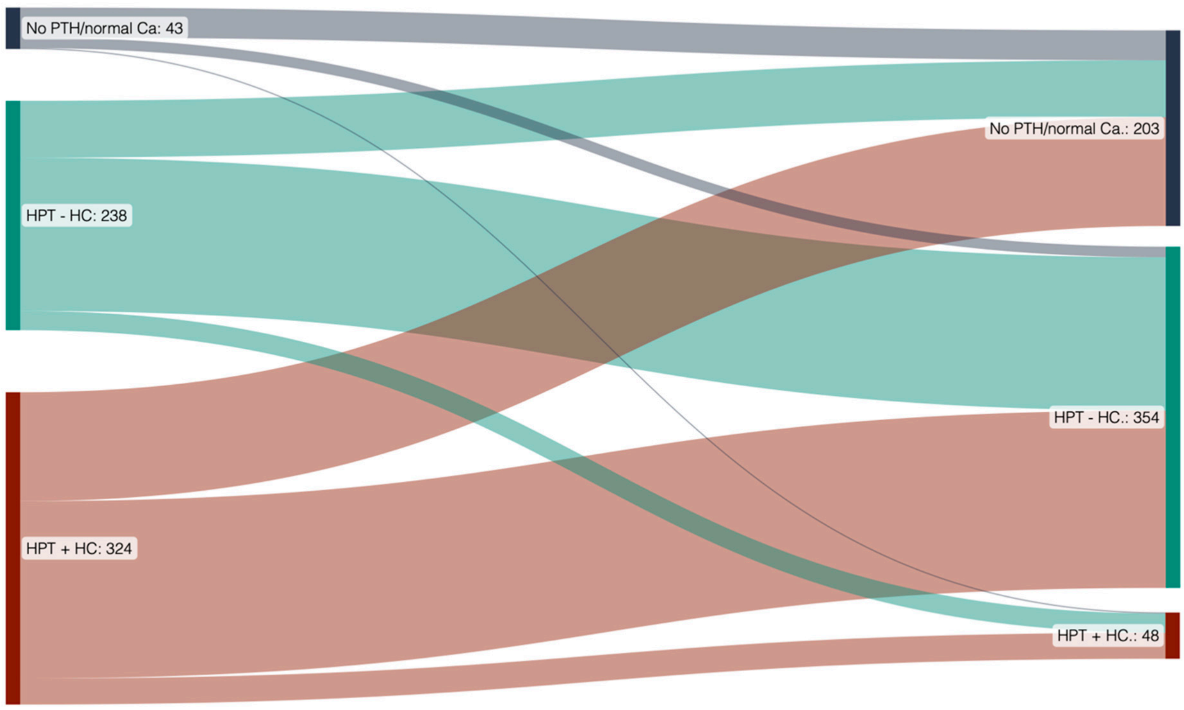

Supplemental Figure S2

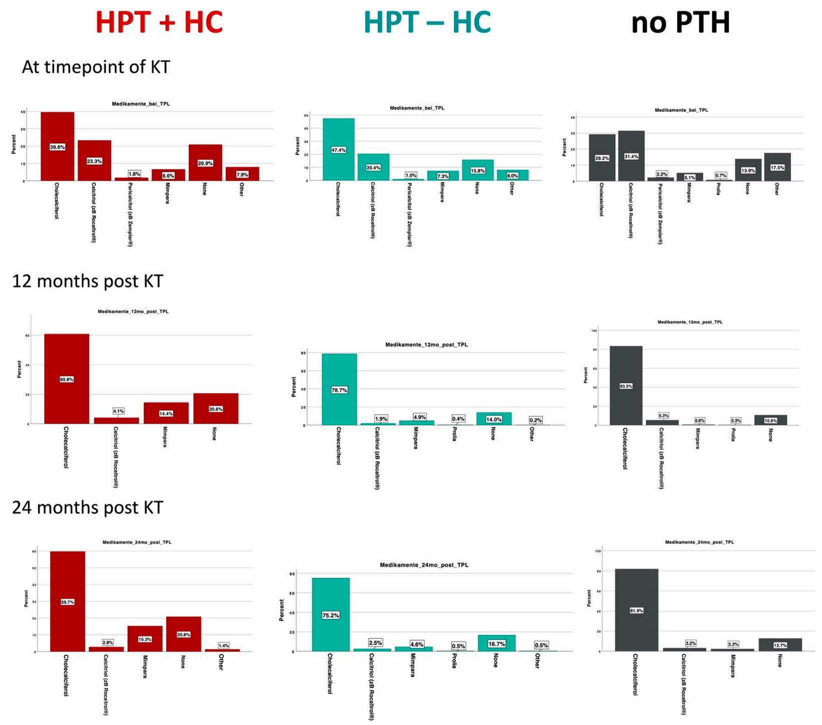

Supplemental Figure S3

Suppl Figure S3A

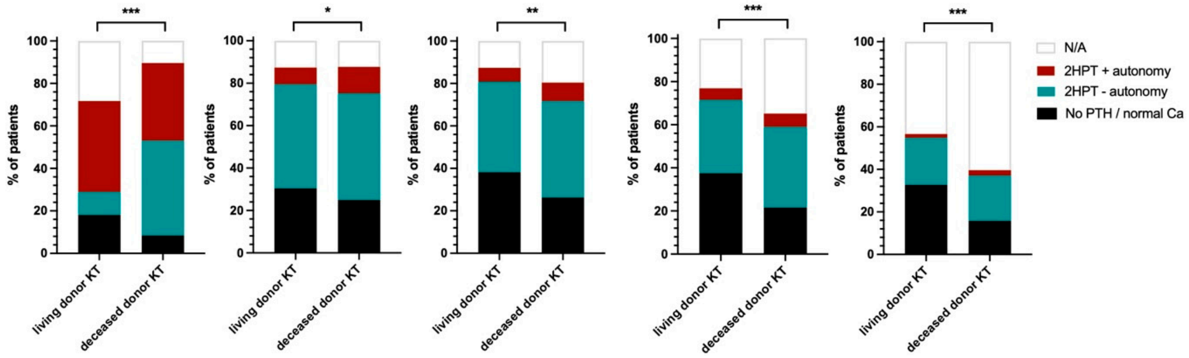

Suppl Figure S3B

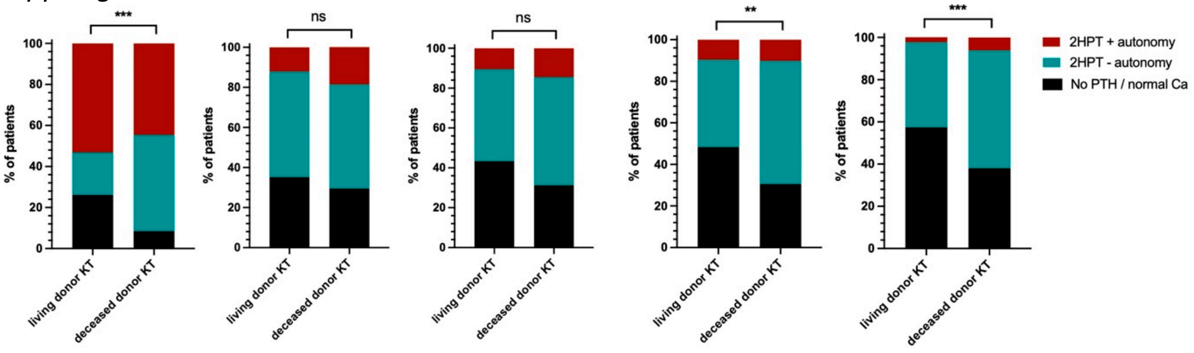

Suppl Figure S3C

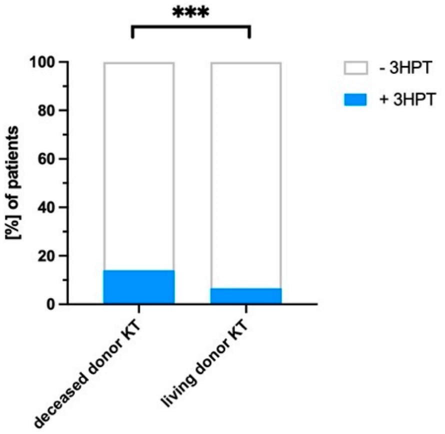

Supplemental Figure S4

Supplemental Figure S4A

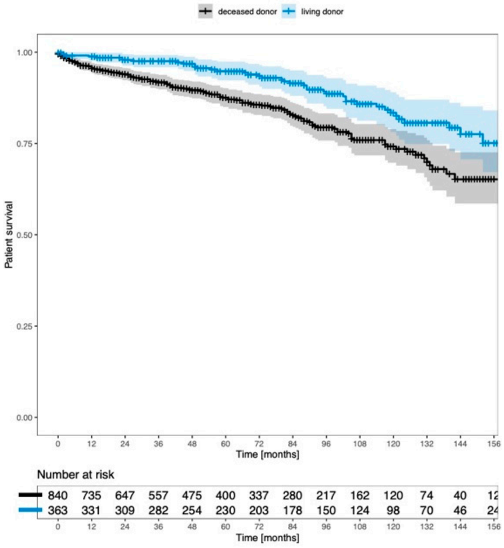

Supplemental Figure S4B

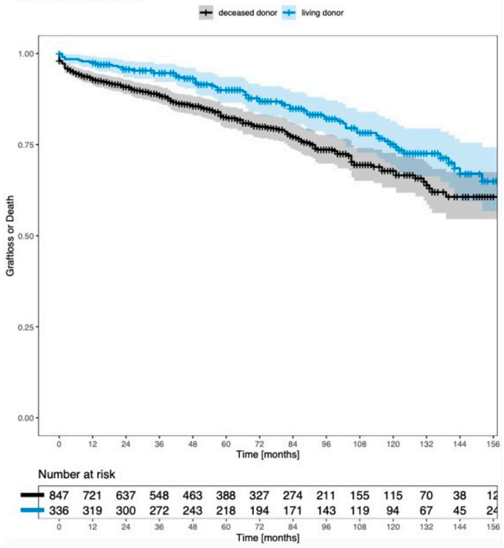

Supplemental Figure S4C

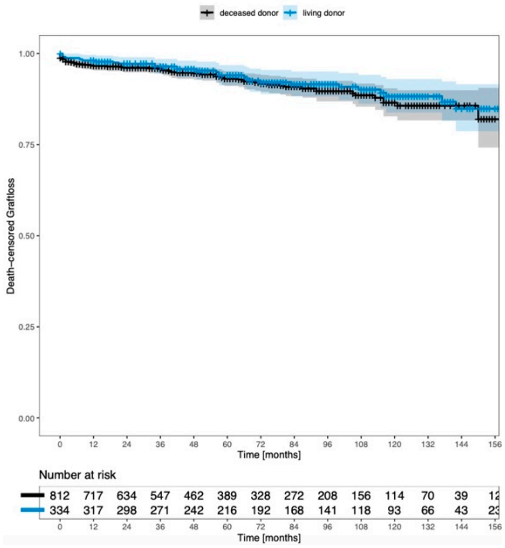

Supplemental Table and Figure Legends

Supplemental Table S1

Effect of pre-transplantation dialysis status on incidence and evolution of hyperparathyroidism and hypercalcemia in KTRs.

Supplemental Table S2

Patient characteristics, and graft- and overall survival for patients with parathyreoidectomy before kidney transplantation compared to the rest of the transplant cohort.

Supplemental Table S3:

Patient characteristics and outcomes of KTRs without hypercalcemia - compared to KTRs with persisting hypercalcemia at 24 month FU or at death (if patients died before). Patient and graft survival data are derived from the Simon-Makuch survival analyses.

Supplemental Figure S1

Sankey diagram of a subset of patients (n=605) with complete data on serum calcium and serum PTH at the two observed timepoints. Left: at kidney transplantation, right: at 24 month follow-up. Diagram created using SankeyMATIC

Supplemental Figure S2

Overview of medical treatment, including Vitamin D, Vitamin D analogs and calcimimetics, at time of transplantation, 12 and 24 months after kidney transplantation.

Supplemental Figure S3

3A: Distribution of no HPT, HPT without hypercalcemia and HPT with hypercalcemia between living donor- and deceased donor KTRs at transplantation and all follow-up timepoints (from left to right: time of transplantation, 6 months, 12 months, 24 months and 60 months after transplantation).

3B: Distribution of no HPT, HPT without hypercalcemia and HPT with hypercalcemia between living donor- and deceased donor KTRs at transplantation and all follow-up timepoints in a subset of patients with complete dataset (from left to right: time of transplantation, 6 months, 12 months, 24 months and 60 months after transplantation).

3C: Incidence of hyperparathyroidism and hypercalcemia over all timepoints between living donor- and deceased donor KTRs.

Supplemental Figure S4:

4A: Overall survival of patients according to donor type. HR:1.93 (95% CI 1.35-2.77), $p<0.001$.

4B: Graft survival of patients according to donor type. HR:1.53 (95% CI 1.14-2.05), $p=0.004$.

4C: Death-censored graft survival of patients according to donor type. HR:1.18 (95% CI 0.76-1.85), $p=0.461$.
